# 3D bioprinting—a model for skin aging

**DOI:** 10.1093/rb/rbad060

**Published:** 2023-06-13

**Authors:** Ryeim B Ansaf, Rachel Ziebart, Hemanth Gudapati, Rafaela Mayumi Simoes Torigoe, Stella Victorelli, Joao Passos, Saranya P Wyles

**Affiliations:** Department of Biology, Colorado State University Pueblo, Pueblo, CO 81001, USA; Mayo Clinic Alix School of Medicine, Rochester, MN 55905, USA; Mayo Clinic Department of Dermatology, Rochester, MN 55905, USA; Mayo Clinic Graduate School of Biomedical Sciences, Rochester, MN 55905, USA; Mayo Clinic Department of Physiology and Biomedical Engineering, Rochester, MN 55905, USA; Mayo Clinic Robert and Arlene Kogod Center on Aging, Rochester, MN 55905, USA; Mayo Clinic Department of Physiology and Biomedical Engineering, Rochester, MN 55905, USA; Mayo Clinic Robert and Arlene Kogod Center on Aging, Rochester, MN 55905, USA; Mayo Clinic Department of Dermatology, Rochester, MN 55905, USA; Mayo Clinic Robert and Arlene Kogod Center on Aging, Rochester, MN 55905, USA

**Keywords:** 3D bioprinting, skin, aging, bioink, regeneration

## Abstract

Human lifespan continues to extend as an unprecedented number of people reach their seventh and eighth decades of life, unveiling chronic conditions that affect the older adult. Age-related skin conditions include senile purpura, seborrheic keratoses, pemphigus vulgaris, bullous pemphigoid, diabetic foot wounds and skin cancer. Current methods of drug testing prior to clinical trials require the use of pre-clinical animal models, which are often unable to adequately replicate human skin response. Therefore, a reliable model for aged human skin is needed. The current challenges in developing an aged human skin model include the intrinsic variability in skin architecture from person to person. An ideal skin model would incorporate innate functionality such as sensation, vascularization and regeneration. The advent of 3D bioprinting allows us to create human skin equivalent for use as clinical-grade surgical graft, for drug testing and other needs. In this review, we describe the process of human skin aging and outline the steps to create an aged skin model with 3D bioprinting using skin cells (i.e. keratinocytes, fibroblasts and melanocytes). We also provide an overview of current bioprinted skin models, associated limitations and direction for future research.

## Graphical Abstract

**Figure:**
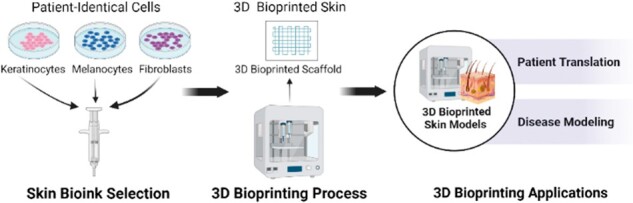

## Introduction

### Need for 3D bioprinting

In the past century, human lifespan has increased by almost 50%, with the projected tripling of the number of persons at 80 years of age or older by 2050 [1]. As adults age, morbidity increases, as does the need for solutions to skin ailments faced by older adults, such as chronic wounds [2], skin cancers [3] and treatments for debilitating skin conditions, such as pemphigoid, unique to the elderly [4]. Regenerative medicine and tissue engineering are areas of growing interest in dermatology due to the accessible nature of the skin. As the outermost organ of the body with a large surface area, intrinsic and extrinsic insults require the skin to regenerate on a monthly basis, which slows with aging [5]. In recent years, 3D bioprinting has gained significant attention from academia and industry as it offers a way to engineer a patient-specific model for diagnostic and therapeutic purposes. The excitement surrounding the potential to bioprint skin can be attributed to many factors, including the availability of high-precision equipment [6], improved understanding of skin morphology and new discoveries surrounding cell manipulation to create organized structures. Additionally, 3D skin bioprinting requires a multidisciplinary approach from experts in material science, engineering, medicine and biology. This technology has enabled 3D bioprinting organoids [7], glands [8] and reproductive tissue [9]. In this review, we will define 3D bioprinting as related to aged skin physiology, outline the biomaterials required to produce an aged skin model and provide direction for future research.

### 3D bioprinting versus 3D printing

3D printing is often used to create 3D structures from materials such as plastics and metals. In various medical specialties, this technology is used to create orthopedic implants, model cancerous tumors by 3D plastic replicas, manufacture medical instrumentation [10] and also used in dental implant planning [11]. In recent years, 3D printing has been utilized in advanced medical applications including surgical planning and drug delivery [10].

3D bioprinting, in contrast, uses biomaterials, cells and culturing techniques to create biologically equivalent models with key features to replicate the phenotype and function of the desired organ. For skin, the key features include cellular architecture, sensation, mechanical strength and elasticity. As skin ages, the microstructure and mechanical properties evolve, and thus an aged 3D bioprinted skin model must closely replicate these changes. Lynch *et al*. [12] conducted a mechanistic analysis of aged skin to determine a decrease in stiffness and elasticity associated with aging along with accelerated thinning in the superficial epidermis and papillary dermis in comparison to the deeper reticular dermis. As such, these changes contribute to many hallmarks of aged skin, such as skin thinning and subsequent easy bruising.

3D skin bioprinting has the potential to uniquely benefit patients. For example, patients with age-related or photodamaged skin thinning need a source of multilayered skin replacement that restores the normal structure and vascular supply. Moakes *et al*. studied the usage of suspended layer additive manufacturing to synthesize tri-layered skin. They showed that 3D bioprinted implants facilitate wound healing at the level of the reticular dermis by having cellular components to remodel the supporting extracellular matrix (ECM). They also observed mobilization of the adipose tissue, which contributes largely to wound repair [13]. Regeneration and adaptability are unique to 3D bioprinting as compared to 3D printing. Furthermore, 3D skin bioprinting provides a novel aged skin model to use for *in vitro* studies.

### Overview of skin structure and function

Replicating an *in vitro* skin model requires an in-depth understanding of skin structure and function. The skin is composed of two main layers: (i) epidermis and (ii) dermis. The epidermis is the outermost portion of the skin and serves as a protective barrier against pathogens as well as damage from the environment. The epidermis can further be divided into the stratum corneum, lucidum, granulosum, spinosum and basale [14]. Keratinocytes, the primary cell type in the epidermis, regenerate in the stratum basale and mature upwards toward the stratum corneum, becoming progressively flattened and anucleated. Keratinocytes are enveloped in cross-linked proteins and lipids that aid in providing the water barrier of the skin through hydrophobic interactions with the environment [15]. Keratinocytes also play a role in calcium absorption by activating cholesterol to form Vitamin D [16]. The deepest portion of the epidermis, the stratum basale, provides UV protection through melanin secretion from melanocytes. The epidermis also plays a role in the innate immune system, with Langerhans cells functioning as antigen presenting cells throughout all layers, but especially in the stratum spinosum [14].

The dermis contains a vast ECM that is primarily composed of collagen, glycosaminoglycans (GAGs), elastic fibers and proteoglycans. The firmness of youthful skin is largely provided by the ECM and the fibroblasts that lie within the dermis to produce collagen [17]. Structurally, the dermis and epidermis are held together by the dermal–epidermal junction (DEJ). This junction interlocks the layers with a dense collagen network to prevent separation and blistering [18].

### Structural and functional changes with skin aging

To develop an aged skin model, baseline understanding of the aging process is needed. Skin aging occurs both intrinsically as a result of metabolic processes, and extrinsically as a result of environmental factors [19]. The progressive sequalae of aging is a result of multiple interdependent pathways termed the hallmarks of aging, which include genomic instability, telomere attrition, epigenetic alterations, loss of proteostasis, deregulated nutrient sensing, mitochondrial dysfunction, stem cell exhaustion, altered intercellular communication [20] and cellular senescence. Cellular senescence is defined as a stable cell cycle arrest that was first described by Hayflick and Moorhead [21]. Although senescent cells do not divide, they remain metabolically active with a pro-inflammatory secretome known as senescence-associated secretory phenotype that contributes to aging [22]. Various factors trigger premature cellular senescence including UV exposure and other environmental triggers [23].

As the skin ages, several changes occur. In young skin, the epidermis borders the dermis in a wavy and undulating pattern, forming rete ridges [24]. With age, the rete ridges and DEJ become flatter due to an alteration in adhesion molecules and loss of dermal papillae that yield a weaker junction between cells. It has been shown that the DEJ is reduced by more than one third in aged skin as compared to young skin, with a decline beginning in the sixth decade of life [25]. These changes in the DEJ contribute to the fragility of aged skin, and leave aged skin more susceptible to tearing, shearing and ulceration.

As skin ages, melanocytes enter senescence as their telomeres shorten [26]. Senescent melanocytes have been found to display elevated p16^INK4a+^ expression (a biomarker associated with cellular senescence) and to secrete CXC chemokine receptor three-dependent mitochondrial reactive oxygen species, which causes a decrease in keratinocyte proliferation [27]. Reduced keratinocyte proliferation contributes to a decrease of epidermal function and integrity, allowing it to be vulnerable to permeation and damage [23]. Langerhans cells also decrease with age, causing a delay in wound healing that result in chronic wounds and infection [28].

In the dermis, aging decreases the number of fibroblasts, macrophages, mast cells and components of the ECM. In wound healing, tissue macrophages play a prominent role in the production of granulation tissue, therefore, a reduction in these cells leads to delayed skin remodeling [28]. In addition, fibroblasts reduce in number due to a limit in their ability to divide as they approach senescence; this concept is described as the Hayflick principle [21]. The reduction in fibroblast number correlates with its function as evidenced by reduced collagen formation, and less fullness in the ECM. Not only is collagen in aged skin reduced, but also it is straighter and more loosely woven, which affects the mechanical properties of skin. These changes to the ECM contribute to dermal thinning and yields an overall less youthful skin appearance [28].

**Figure 1.**
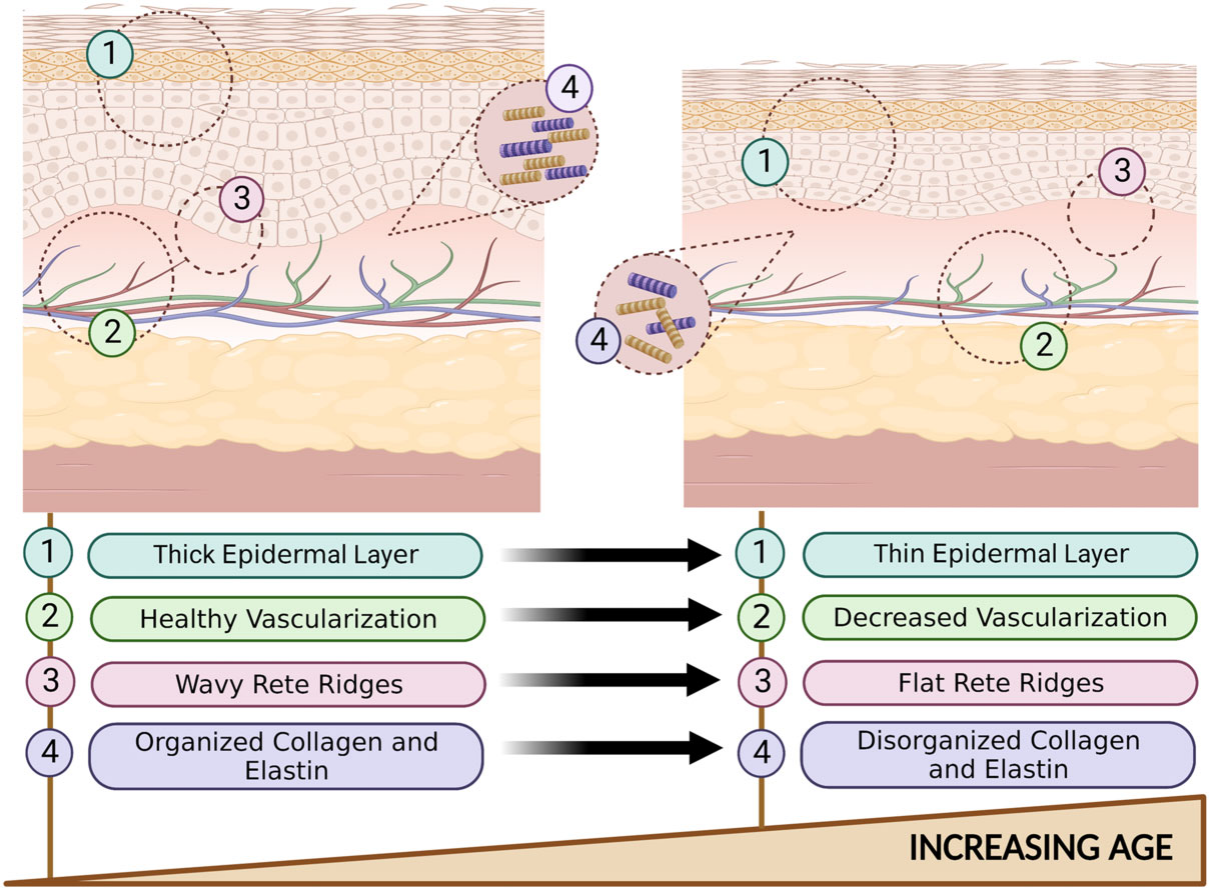
Histological changes in skin aging—young skin versus old skin.

Finally, as skin ages, the number of blood vessels that supply the skin is also reduced [29]. In healthy skin, a complex microvascular network supplies skin cells with oxygen and energy needed for metabolism, regeneration and wound healing. A decrease in vasculature results in susceptibility to chronic wounds and markedly reduced ability for cells to regenerate [30]. Figure 1 summarizes these changes.

### Current models to study aging

In recent years, there have been significant advancements in the development of an aged skin model. For example, the INK-ATTAC transgene mouse model that is capable of elimination of p16^INK4a+^ positive cells associated with aging skin phenotypes [31]. The creation of this model allowed for advancements in research corresponding to cellular senescence and aging in skin, however further progress is needed in studying the progression of aging in human skin. Although skin has well-recognized regenerative properties, the parameters of skin physiology, such as skin structure and age-related DNA damage, in addition to hair-bearing sites significantly vary across human skin compared to the rodent skin model [32]. Therefore, development of a 3D bioprinted model is essential to reliably simulate the progression of aging.

Studies describing the development of aged skin models are limited by the variability in the methods utilized to create such model. By using a 3D culture, Jeong *et al*. developed an aged full-thickness skin model by using mechanical stimulus and the Circadian rhythm. While most *in vitro* models of skin experience contractions during incubation, this model was cultured for 28 days and used a photo-crosslinker with a stimulated Circadian Rhythm environment to mimic the aging process [33]. Yet, this model cannot be used as a holistic model of aged skin due to the lack of major skin components like immune cells, capillaries, hair follicles, peripheral nerve cells and sweat glands [33]. 3D bioprinting skin models, in contrast, have achieved results closer to the structure of native skin including the creation of a skin substitute that contain sweat glands and hair follicles [8].

### 3D bioprinting to model aged skin

#### Regeneration and wound healing

Aging skin pathologies have limited root-cause targeted solutions. For example, elderly patients are at increased risk for skin cancer [3], which may require advanced reconstruction with Mohs micrographic surgery [34]. One in four non-melanoma skin cancers are treated with Mohs micrographic surgery [35], including dermatofibrosarcoma protuberans, atypical fibroxanthomas, extramammary Paget disease, Merkel cell carcinoma, microcystic adnexal carcinoma, sebaceous carcinoma [36] and basal cell carcinoma, which represents 80% of all skin cancers [37]. However, Mohs micrographic surgery carries limitations both functionally and cosmetically, including suboptimal scarring and tissue necrosis. Scarring complications can result from a mismatch in flap thickness to the donor site, and the necrosis complications result from a flap with poor vascularization [38]. Development of 3D bioprinted flaps that closely replicate excised tissue with innate vascular supply could revolutionize reconstructive surgery and offer a means to prevent current complications [7].

3D bioprinting also carries potential to provide a solution to blistering skin diseases that may require the transplantation of skin grafts. Relevant conditions include pemphigus vulgaris, an autoimmune disease that affects desmosomes connecting the keratinocytes to each other [39], and bullous pemphigoid, a blistering disease in elderly patients that results in antibody-mediated tense subepidermal blisters and subsequent dermal-epidermal separation [40]. If medical management is not sufficient and severe blistering results in full-thickness skin loss, then split-thickness skin grafting is the gold standard of treatment [41]. If left untreated, this condition has potential to become life threatening [4].

Skin grafts can be utilized for various wound closures whereby primary intention is not an option, or in cases where applying tension leads to a poor cosmetic outcome or loss of mobility due to contracture. If a wound is located in an area where functional mobility and cosmetic outcome is preferred, such as the hand or face, then a wound larger than 3 cm requires a skin graft [42]. Skin donor availability is the major limitation. Cosmetically, the current harvesting process causes scarring, leaving a mesh-like pattern on the grafted site once healed [41]. Furthermore, careful attention must be paid to match the thickness of the graft skin to the thickness of the recipient area, as a mismatch in thickness can create an indentation or a bulge postoperatively [43]. Functionally, both the graft and donor site are at risk for infection as well as post-procedural pain. A major disadvantage to the current use of split-thickness skin grafting is that the open mesh-like pattern that causes the healing process to occur through secondary intention [41]. A recent study by Baltazar *et al*. [44] describes a 3D bioprinted skin substitute, which eliminates the need for skin grafts in mice. This engineered multilayered graft utilized human endothelial cells and human placental pericytes, which self-assembled *in vitro*, to form microvascular networks that demonstrated perfusion *in vivo* when implanted into the mouse dorsum. This model contained fibroblasts and endothelial cells suspended in collagen as well as keratinocytes from human foreskin as the epidermis. It is postulated that such a skin substitute not only eliminates the need for graft harvest but also improves wound healing due to better site-specific conformity and increased vascularization [44]. 3D bioprinting of skin grafts also ensures that the graft precisely matches the architecture of lost skin and allows for the potential for scarless wound healing [45].

#### Disease modeling, treatments, and drug testing

3D bioprinted skin grafts can also be used in the treatment of diabetic ulcers. Type II diabetes mellitus is a chronic condition characterized by high blood glucose and insulin resistance that affects many older adults [46]. Common complications of type II diabetes mellitus include development of foot ulcers [47] that affect 19–34% of all diabetics across their lifetime, significantly increasing both morbidity and mortality [48]. These wounds become chronic and prone to infection due to lack of perfusion, sometimes lasting months to years. Diabetic foot ulcers may require skin grafts; however, these grafts often fail to heal due to a lack of vascularization in the implanted tissue. To address the lack of vasculature in allogenic skin grafts, Yanez *et al*. utilized 3D bioprinted full-thickness skin grafts with a microvascular network printed with human endothelial cells. These grafts were used to close full-thickness wounds in athymic mice. Between days 14 and 16 after implantation of the skin graft, each graft became integrated into the mouse and was found to resemble the surrounding tissue. All mice healed without infection [49].

Another important and versatile use of 3D bioprinting is the possibility to perform drug testing on 3D bioprinted human skin equivalents, improving the current pre-clinical animal model or 2D cell culture models. Animal testing can be cost-prohibitive, associated with ethical concerns and lacks human correlation, especially considering the variability in age physiology. For example, the rat rodent model is often used in drug testing; however, rats reach sexual maturity at six weeks old, and in adulthood, every month of rat aging is equivalent to 2.5 years of human aging, yielding it impractical to replicate human skin aging in rodents. Further, murine skin contains a layer of striated muscle that allows the skin to move independently from the deeper layers, providing more rapid wound approximation than human tissue [50]. As such, murine models lack human specificity, especially regarding the aged human skin and wound healing models [51]. Bioprinting human skin equivalents not only eliminates the cost and variability of animal testing [52], but also serve as a replacement to human subjects [53]. Currently, there is no viable alternative to animal and human testing. 2D *in vitro* studies are conducted on cell culture plates, but this model is distinctly different from the environment of *in vivo* studies and presents confounding effects [54]. 3D bioprinting is an ideal solution to the limitations of animal testing, human testing, and 2D models.

## Skin bioprinting

### Steps of 3D bioprinting skin

**Figure 2.**
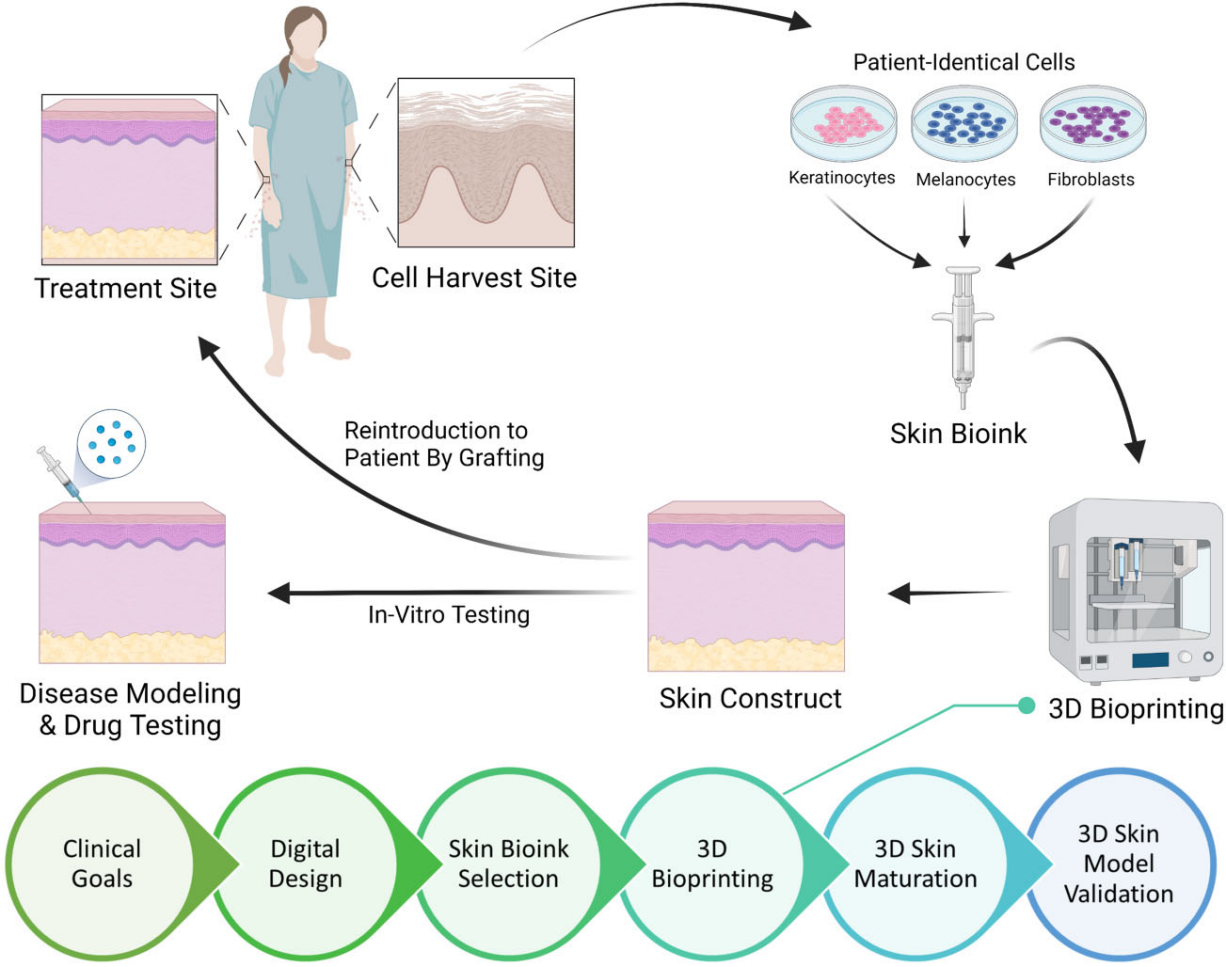
Process of 3D bioprinting human equivalent skin models.

The process of skin bioprinting occurs in six main steps, as represented in Fig. 2. The first step is the determination of clinical goals, where the researcher establishes clinical and research goals aligned with a design criterion for the project based on pre-existing models, diseases and real-life structures to emulate. The second step is the usage of a 3D computer-aided design software and a computer-aided manufacturing software to produce 3D digital models and generate tool path, respectively [55]. Then, bioink selection, a critical step in the bioprinting process, requires background research about the material properties of the chosen bioink as well as the biochemical and physiological environment of the cells to make the most appropriate choice. The fourth step is the printing process, which requires a 3D bioprinter, printheads, and often temperature control and sterile environments. This step includes an optimization sub-step, mainly for parameter adjustment, to achieve high resolution. The fifth step is the functionalization step, where the printed model is placed into an incubator or bioreactor, allowing the cells to gain functionality, stability, and growth. The sixth and final step is to validate the model for its intended application. Typical applications include *in vitro* testing, disease modeling or *in vivo* implementation [55].

### Design consideration for a generalized skin bioink formulation

**Figure 3.**
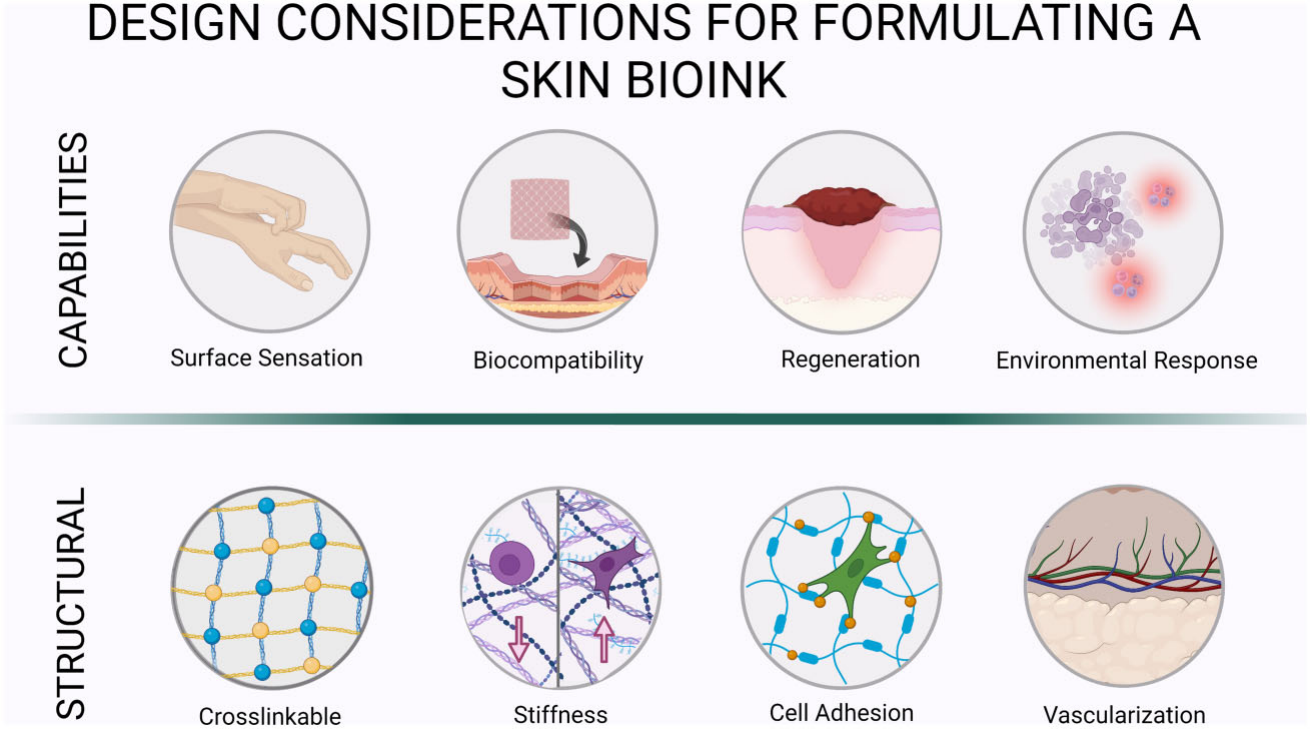
Design characteristics of an ideal skin bioink.

Biopolymers are often used in modeling soft skin tissue, especially as scaffolds, due to their high degree of biocompatibility and biomimetic properties that allow for native tissue regeneration [56]. For skin tissue, the ideal biomaterial provides a mixture of the following functions (Fig. 3):

#### Surface sensation

Surface sensation remains the least explored properties of native skin in 3D bioprinting, one of the most desired assets [55]. In the skin, sensation uses complex topographically distributed receptors such as the mammalian cutaneous receptors, which have not been explored well with 3D bioprinting [57]. Other studies have reported that biopolymers used in 3D bioprinting scaffolds, such as collagen, gelatin, alginate, hyaluronic acid, dextran and fibrin [58], cause a loss of sensation in the grafted area. Surface sensation in skin provides a challenge since most materials—especially polymers, undergo polymeric surface modification to enhance biocompatibility by improving functionalization of cell adhesion motifs such as Arginylglycylaspartic acid (RGD), however, this leads to the loss of sensation due to the new polymeric reactions, as it impacts surface sensory response and growth of the sensory nerves [59].

#### Mechanical structure and properties

For deposited skin constructs, scaffolds are necessary to mimic the mechanical structure and properties of the native tissue ECM. Cellular responses are optimal at a scaffold stiffness (elastic modulus) that is reminiscent of native tissue ECM and stiffness has shown to direct diverse cellular responses including cell differentiation [60, 61]. Collagen is an optimal candidate for skin scaffold as it is a natural constituent of native skin, exhibiting mechanical properties of the native ECM while providing native cell adhesion ligands. However, liquid pre-polymers of collagen, which are derived from animal sources do not polymerize instantaneously and the polymerized collagen has a low mechanical strength, hindering 3D bioprinting of skin at the cellular level resolution. Although biopolymers such as alginate and synthetic polymers such as poly(ethylene glycol) polymerize rapidly and offer superior mechanical properties, they are inferior to collagen in replicating the native ECM structure and properties. Hence, collagen and its derivative gelatin have been widely used for formulating skin bioinks [62]. Ideally, the mechanical properties of the bioprinted skin post-maturation should align with the key parameters of successful biomedical applications summarized as strength, stiffness, compressive strength, wear and creep resistance [59].

#### Permeability and solubility

Since the skin serves as a regulator of endocrine and exocrine functions, a 3D bioprinted model necessitates its ability to maintain homeostasis with water contact. For example, the skin contributes largely to water regulation due to the hydrophobic layer interactions at the plasma layer; as such, a 3D bioprinted model should be capable of adequate water regulation [63].

#### Environmental response

The biomaterial used for skin should have strong water–biomaterial interactions that modulate cell adhesion. Without cell adhesion, the bioink may be at risk of immediate rejection due to biocompatibility issues with grafted site [64]. Additionally, environmental response to various environmental cues such as temperature are important for thermoregulation and maintaining homeostasis in the body, as well as protection from pathogens.

In summation, the properties mentioned above are important for an optimal skin bioink. Table 1 offers a summary of current representative bioinks for 3D bioprinting skin with their associated advantages and disadvantages.

**Table 1.** Representative bioinks for 3D bioprinting of skin

Bioink types	Overview	Cells used previously	Advantages	Disadvantages	References
Collagen: BD BioSciences Advanced BioMatrix Vitrogen Flexcell Corning	Structural component of skin and connective tissue	NIH-3T3 fibroblasts Human keratinocytes cell line (HaCaT) Fibroblasts (HEF-1) Neonatal epidermal keratinocytes	Mechanical properties similar to native skin, enzymatically degradable and contains native cell adhesion ligands	Acid soluble	[44, 49, 59, 62, 65–70]
Gelatin: Sigma-Aldrich CELLINK^®^	Protein formed by collagen hydrolysis	Keratinocytes Fibroblasts	Highly biocompatible, high water solubility, thermally reversible gelation	Low shape fidelity and rigidity	[71–74]
Fibrin: Baxter Johnson & Johnson Sigma-Aldrich	An insoluble protein used for blood clotting and sourced from human plasma	Keratinocytes Fibroblasts	Rapid gelation, biologically relevant, limited printability, enzymatically degradable	Limited printability, low mechanics limit utility	[65, 75]
Alginate: NovaMatrix-3D PRONOVA Sigma-Aldrich	Sourced from brown algae and must be modified with adhesive ligands for cell attachment	Neonatal human foreskin fibroblasts NIH-3T3 fibroblasts	Ionic crosslinking enables cell encapsulation	Covalent crosslinking required for strength	[65, 75]
Chitosan	Polysaccharide obtained by fungal fermentation or outer skeleton of animals	NIH-3T3 fibroblasts Human keratinocytes cell line (HaCaT)	High compatibility, antibacterial properties	Slow gelation rate	[76]
Hyraluronic acid (HA)	Non-sulfated GAG present in connective, epithelial and neural tissues	Combined with collagen, fibroblasts, keratinocytes and melanocytes	Rapid gelation, cell proliferation promoter	Low stability	[65]

### Design considerations for an aged skin bioink formulation

#### Ability to express cellular senescence

Although there is limited research regarding cellular senescence in 3D bioprinting, a review article by Al Shobul *et al*. [77] explored the role of 3D bioprinting and senescence in cancer therapy. There is more research focusing on display of cellular senescence in 3D models. For example, Pauty *et al*. explored the creation of a 3D tissue modeling for studying the effects of senescent fibroblasts on blood vessels. Other researchers have explored senescence in 3D human cartilage [78] and 3D and 2D cultured adipose-derived mesenchymal stem cells [79]. Other 3D models for cellular senescence are highlighted in Milligan *et al.* [80] reviews.

#### Tunable properties

Because aging skin is so unique compared to young skin, the model can benefit from a tunable adjustability of its properties. Jian *et al*. proposed tunable mechanical properties with elastic moduli 4–62 kPa and controlled biodegradability using supramolecular hydrogels self-assembled from short peptides [81]. With this model, the ideal natural environment for a variety of cells and organs, including skin, can be met. For an aged skin model, the mechanical modulus can be adjusted to be lower as seen with natural aging.

## Discussion

### Advantages and disadvantages of 3D bioprinting

3D bioprinting portends several advantages including the ability to fully recreate a functional human skin equivalent, eliminating the need for animal testing [52]. The availability to recapitulate patient-specific tissues that are personalized to match the graft site during reconstructive surgery also offers the potential to decrease or eliminate post-procedural scarring [45]. With scarce available donor sites in patients with blistering skin conditions, such as bullous pemphigoid and pemphigus vulgaris, the 3D-bioprinted skin grafts for such conditions eliminate the need for donor sites and the related complications [41].

The 3D-bioprinted skin also offers the advantage of the relatively short duration required to manufacture the printed product. Traditionally, a human plasma-based bilayered skin generated for burn treatment and surgical wounds required an estimated three weeks for production [75]. With 3D bioprinting, a human bilayered skin using bioinks containing biopsy-obtained plasma, fibroblasts and keratinocytes could produce 100 cm^2^ area of bioprinted skin in <35 min [75]. For burn patients, time to intervention and skin grafting are vital to limit scarring, infection and further complications [75]. Additionally, autografts and other commercially available skin equivalents are often limited in size. Large injuries introduced by motor vehicle accidents and military operations endanger patient or active-duty military survivability due to the amount of time and resources required to cover and stabilize the wound. For *in situ* burn wound repair, studies have shown that an extrusion-based bioprinting method could precisely deliver skin cells to the wound, and closure was observed within 3 weeks in a nude mouse wound model, showing organization of skin cells, dermal collagen and a fully developed epidermis [82].

Advanced 3D bioprinting technology has promising potential to offer off-the-shelf storage requirements for healthcare centers and hospitals. A handheld skin printer was created to enable the *in situ* formation of skin tissue sheets of various architectural compositions. This technology can produce consistent sheet formation by coordination of flow rates; as a result, the possibility of scarring is reduced with this 3D bioprinted skin intervention [65].

Mechanically, with the different bioprinting strategies presented in Table 1, 3D bioprinted skin can appear more natural than traditional skin grafts. For instance, studies utilizing 3D bioprinting melanocytes offer potential for 3D bioprinting skin to look natural and represent various skin tones, as the pigmentation can be altered by changing the concentration of melanocytes [67]. The collagen levels in the 3D-bioprinted skin have potential to undergo alterations to appear more youthful, as there is an association between high collagen and youthful, healthy skin [83]. This is of interest in plastic surgery and cosmetic dermatology.

**Figure 4.**
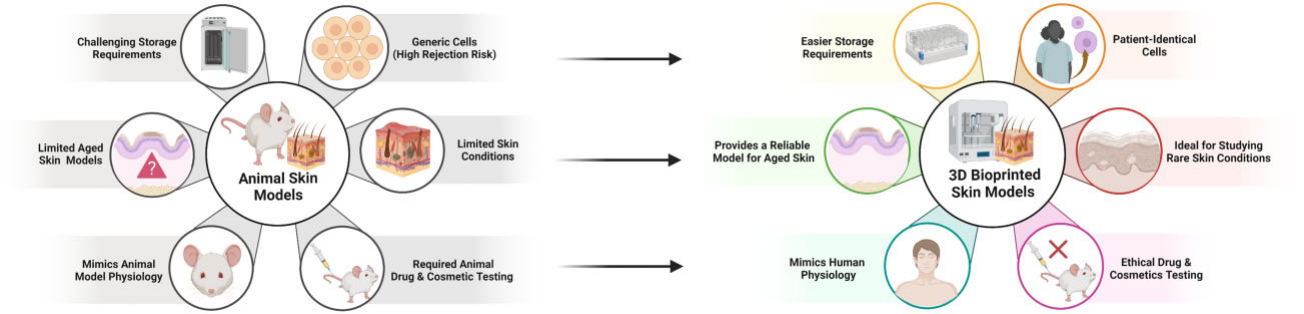
Advantages of 3D-bioprinted skin models compared to animal models.

3D bioprinting further provides a patient-specific and customizable therapeutic option that is highly biocompatible due to the usage of patient cells. Previous studies regarding artificial skin graft rejections have shown that the rate of rejection is increased based on the graft volume and antigen incapabilities between the donor and the patient [84]. In this case, 3D bioprinting can address this problem by allowing researchers to selectively choose donor cells that avoid immune rejection. Figure 4 summarizes the advantages of 3D bioprinted skin models when compared to animal models.

Several limitations regarding 3D bioprinting exist. The main disadvantage is its manufacturing process and resources that may not be readily available in all facilities. In addition to accessibility, 3D bioprinting also has functional limitations. For instance, ongoing research into methods for creating functional nerves within a tissue scaffold is warranted [85]. 3D bioprinting has yet to make fully functional nerves to innervate tissues [86], which is an important characteristic of skin.

Certain skin biomaterials also harbor disadvantages. For example, collagen-based biomaterials used in 3D bioprinted skin have poor printability and long crosslinking time, rendering a challenge to the creation of 3D constructs with predetermined shape and configurations. In contrast, materials used in 3D non-bioprinting are more efficient at printing predetermined configurations such as a hierarchical truss structure [87]. New bioinks are continuously being developed for bioprinting, which address the setbacks encountered with collagen bioinks. A recent study optimized a polyelectrolyte gelatin–chitosan hydrogel biomaterial for 3D bioprinting at room temperature to achieve high shape fidelity and good compatibility with fibroblasts [76]. Thus, 3D bioprinting skin carries exciting new potential in the field of regenerative medicine, dermatology and advancement for the treatment of age-related skin conditions.

### Future directions

This review highlights the advent of 3D bioprinting skin technology and its applications in the aging skin model. Although favorable bioink characteristics were discussed, several factors are still under development. Current 3D skin models represent a full-thickness structure that lacks immune cells, vascularity, nerves and sweat glands [86]. Despite the partial clinical success story of 3D bioprinting skin, there are increasing demands from both patients and clinicians to address the existing limitations such as improved surface sensation and regeneration [88]. A major challenge that remains in creating skin bioinks is the ability to have the sum of these factors within a single model.

3D bioprinting is often projected as a high-throughput method for fabricating skin grafts in future applications since it uses the patient cells, providing a compatible substitution for skin grafts, and a reduced chance of bodily rejection [41]. Yet, a challenge that comes with this task is being able to create a universally compatible bioink for minimizing treatment time. Given that 3D bioprinting requires optimization at the printing stage, the time frame to build a successful model is a major drawback as opposed to standard-of-care procedures for skin grafts and commercially available skin substitutes like Apligraf [89].

Additionally, with aging skin models, the cellular senescence concept is still being researched [31]. A solid foundation of the hallmarks of skin aging, including cellular senescence, is vital to recapitulate age-related skin changes. Both fields are relatively new and require further investigation before an optimal model is achieved.

## Conclusion

Advancements in 3D bioprinting make the prospect of developing a human skin aging model an attainable future goal. The ability to 3D bioprint skin aids in the recapitulation of human aged skin conditions and revolutionizes skin grafts, as well as dramatically improves reconstructive surgical outcomes, both aesthetically and functionally. Future research could explore the methods for 3D bioprinting full-thickness skin aging using available bioinks with emphasis on attaining the native features of human skin and adapting them to the multifactorial aging process.
